# DNA Damage and Bisphenol Levels in Chronic Kidney Disease Patients Undergoing Hemodialysis

**DOI:** 10.3390/jox15050167

**Published:** 2025-10-17

**Authors:** Cesar Emilio Ruiz, Lourdes Vela, Martí Nadal, Neus González, Ricard Marcos, Alba Hernández, Susana Pastor, Elisabeth Coll

**Affiliations:** 1 Nephrology Department, Fundació Puigvert, 08025 Barcelona, Spain; cruiz@fundacio-puigvert.es; 2 Group of Mutagenesis, Department of Genetics and Microbiology, Faculty of Biosciences, Universitat Autònoma de Barcelona, 08193 Cerdanyola del Vallès, Spain; lulis002@hotmail.com (L.V.); ricard.marcos@uab.es (R.M.); alba.hernandez@uab.cat (A.H.); 3 Faculty of Health Sciences Eugenio Espejo, Universidad UTE, Quito 170508, Ecuador; 4 Laboratory of Toxicology and Environmental Health, School of Medicine, Universitat Rovira i Virgili, 43201 Reus, Spain; marti.nadal@urv.cat (M.N.); nieves.gonzalez@iispv.cat (N.G.); 5 Institut d’Investigació Sanitària Pere Virgili (IISPV), 43204 Reus, Spain

**Keywords:** bisphenols, chronic kidney patients, hemodialysis, BPA-free dialyzers, genotoxicity, comet assay, micronucleus assay

## Abstract

Bisphenol (BP) compounds are widely present in the environment, primarily due to their use as plastic additives. These substances involve health risks, particularly as endocrine disruptors. While the general population is chronically exposed, patients with end-stage chronic kidney disease undergoing hemodialysis (HD-CKD) represent a particularly vulnerable group. This is due to both impaired renal clearance of circulating BPs and potential contamination from plastic-containing dialyzers used in extracorporeal blood circulation. In this longitudinal study, from the 35 HD-CKD patients initially selected, 25 changed their conventional dialyzers to BPA-free dialyzers for 6 months. Blood serum samples were collected, at baseline and after the intervention, to quantify levels of five BP analogues: Bisphenol A (BPA), Bisphenol AF (BPAF), Bisphenol F (BPF), Bisphenol B (BPB), and Bisphenol S (BPS). Genotoxicity was assessed using the comet assay and the micronucleus test on peripheral white blood cells. Among the analyzed BPs, only BPAF showed a statistically significant reduction when using BPA-free dialyzers. In terms of genotoxicity, a significant decrease was observed only in primary DNA damage (mainly DNA strand breaks), with no notable changes in chromosomal damage. This is the first study to detect multiple BP analogues in HD-CKD patients, beyond BPA, and to associate human exposure to BPs with DNA damage biomarkers. The observed reduction in DNA damage in parallel with decreased BPAF levels highlights the importance of monitoring and minimizing BP exposure of this high-risk population.

## 1. Introduction

Chronic kidney disease (CKD) is a major global health issue with significant socioeconomic impact. Over the past few decades, its prevalence has risen markedly, likely due to increased survival rates from chronic conditions such as diabetes mellitus and hypertension, as well as an aging population. It is currently estimated that approximately 850 million people worldwide are affected by some stage of CKD, with two-thirds of these individuals in advanced stages [1]. The progressive decline in kidney function leads to a range of systemic effects that collectively define the clinical syndrome of CKD. Once end-stage renal disease (ESRD) is reached, renal replacement therapy becomes necessary, with hemodialysis (HD) being the most initiated [2]. HD is a technically complex extracorporeal blood purification method that has experienced improvements in recent decades by using technology that increases the removal of uremic solutes and by using more biocompatible materials that improve hemocompatibility. However, many challenges and unanswered questions remain. Patients with advanced-stage CKD undergoing HD experience a combination of endogenous factors (such as oxidative stress, uremic toxins or chronic infections) and exogenous factors (exposition to membranes and dialysis fluids) that promote a state of chronic inflammation. This contributes to accelerated aging and is associated with high morbidity and mortality, primarily due to cardiovascular factors [3,4]. Among these factors, the occurrence of microplastics is of particular concern [5]. During hemodialysis, circulating blood comes into direct contact with polymer-based medical devices, including tubing and dialysis membranes. This interaction can promote the release of microplastics, as well as the leaching of plastic additives and other chemical constituents [6,7,8,9].

The presence of microplastics in the human urinary tract has been already reported, suggesting their involvement in kidney pathologies via inflammation and alteration of mitogen-activated protein kinases signaling pathways [10]. Although reports of microplastics in human samples have increased in recent years, the lack of standardized and widely accepted methodologies for their detection and quantification introduces considerable uncertainty in the reported concentrations. This limitation is particularly critical for nanoplastics, where the accuracy and reliability of measurements across human body compartments remain highly questionable. Accordingly, the identification of plastic additives in human biological samples is suggested as a surrogate biomarker of microplastics exposure levels [11]. Among these plastic additives, bisphenols (BPs) stand out. BPs are additives used in plastic manufacturing to enhance hardness, transparency, durability, and lightness [12]. BPs are one of the most widely produced synthetic substances worldwide, mainly associated with the plastic industry, due to their advantages as plastic additives. Consequently, their presence has been noted in water, soil, and air all around the world [13,14,15].

A range of analytical techniques can be applied to quantify bisphenol A (BPA) and its analogues, including high-performance liquid chromatography (HPLC), immunoassays, and gas chromatography–mass spectrometry (GC-MS) [16,17,18]. In this study, we selected GC-MS for several reasons. First, our laboratory had previously developed and validated a protocol for detecting bisphenol analogues in blood samples using this technique [19]. Second, GC-MS offers excellent chromatographic resolution and generates reproducible mass spectra that can be directly matched to reference libraries. These characteristics minimize matrix interferences and enhance sensitivity, particularly for compounds that are suitable for derivatization [20]. Finally, although GC-MS typically requires more extensive sample preparation than liquid chromatography–mass spectrometry (LC-MS), it remains a cost-effective and robust method, supported by well-established standardized protocols [16]. Humans are certainly exposed to BP as demonstrated in a recent work reviewing studies published between 2000 and 2023. Results reported the widespread occurrence of environmental exposure and, most relevant, a tendency to increase exposure levels over time [21]. In humans, CKD patients are specifically sensitives to BP exposure since the primary route of BP elimination is through the kidneys, CKD patients (and particularly those undergoing HD) are more susceptible to its accumulation. In addition, because the HD procedure itself involves direct blood contact with plastic components of the extracorporeal circuit that may contain BPs, its migration into the bloodstream can occur due to conditions such as heat and friction that facilitate its release. Accordingly, its presence in CKD patients has been already reported [22] and measures to reduce such exposure have been proposed [23] due to their inability to excrete such pollutants and the presence of BP in some dialysis related devices [22,24].

Toxicity studies on bisphenol A (BPA) have demonstrated adverse effects on the reproductive, nervous, immune, metabolic, and cardiovascular systems, as well as association with cancer development and albuminuria [25,26,27]. Other analogues such as Bisphenol AF (BPAF), Bisphenol F (BPF), Bisphenol B (BPB), and Bisphenol S (BPS) have been studied showing their role as endocrine disruptors, with obesogenic effects and producing reproductive diseases or also promoting certain cancers [28,29]. Thus, BPA was the main analogue determined in polysulfone membranes, while BPS was detected in polyethersulfone membranes [22].

One of the potential harmful effects associated with BP exposure is genotoxicity. This is mainly due to the well-known strong association that exists between DNA damage induction and drastic health consequences for humans [30]. The association of CKD incidence with genomic damage was confirmed in a large study involving a total of 602 subjects in different stages of the progression of pathology. CKD patients had significantly higher levels of DNA damage than controls, but no significant differences were observed between stages, indicating genomic instability as a characteristic of CKD patients [31]. In addition, since a positive relationship between genomic damage and all-cause mortality was demonstrated, these results pointed out that CKD patients with high levels of DNA damage are indicative of a poor prognosis in HD patients [31].

This study is the first to evaluate a broad spectrum of bisphenol analogues in patients with chronic kidney disease (CKD) undergoing hemodialysis (HD), while examining the association between bisphenol exposure and DNA damage. In addition, the potential protective role of BPA-free dialyzers in mitigating genotoxic effects was investigated.

## 2. Materials and Methods

### 2.1. Study Population

Thirty-five patients undergoing chronic hemodialysis at the Puigvert Foundation in Barcelona were included in the study. The Puigvert Foundation (IUNA) is an internationally renowned specialized center within the Hospital de Sant Pau in Barcelona and affiliated with the Autonomous University of Barcelona (UAB). From a clinical perspective, 12,000 patients with CKD are seen in the Nephrology Department each year.

Inclusion criteria were informed of consent, age over 18 years, with a dialysis vintage longer than three months. Exclusion criteria included patients undergoing online hemodiafiltration (type of hemodialysis with high convective volume), incremental dialysis (less than 3 times per week), and those with severe inflammatory or neoplastic conditions or an expected survival shorter than the study follow-up period. All participants were treated with the same type of hemodialysis, prior to the study, with low-flux hemodialysis, three times per week, for an average of 3.5 to 4 h per session, using a low-flux dialyzer, with a polyethersulfone membrane and a polycarbonate housing (VitaPES^®^, Palex Medical, Barcelona, Spain).

All participants provided written informed consent, and blood sample collection was conducted in accordance with protocols approved by the Ethics Committee of the Puigvert Foundation, adhering to the principles outlined in the Declaration of Helsinki. The certificate number for the approval was FP: 2021/02c.

### 2.2. Study Design

This was a prospective interventional study in which each patient served their own control over a 6-month follow-up period. Clinical, laboratory, and demographic data were collected at baseline. For each patient, blood samples were taken before the dialysis session (First sample) to assess baseline BP levels in blood serum and DNA damage after a period of time using conventional dialyzers. Following this initial assessment, the dialyzer was replaced by another one of similar specifications (low flux dialyzer with a polyethersulfone membrane) but with a bisphenol-free housing (ELISIO^®^, Nipro, Osaka, Japan), while maintaining the same dialysis regimen, except for routine clinical adjustments. Participants continued with regular dialysis sessions using the BPA-free dialyzer for a 6-month follow-up period. During this time, 10 patients dropped out of the study due to various reasons, such as death, kidney transplantation, or relocation. In the 25 patients who completed the study, final laboratory data were collected, and a second assessment of bisphenol levels and genotoxicity was conducted.

### 2.3. Determination of BP Levels in Blood

#### 2.3.1. Instrumentation

This analysis was performed using a 7890A gas chromatograph (GC) coupled to a triple quadrupole mass spectrometer (QqQ) series 7000, both from Agilent Technologies (Santa Clara, CA, USA). Compound separation was carried out using an HP-5MS UI chromatographic column (30 m × 0.250 mm × 0.25 μm) (Agilent Technologies, Santa Clara, CA, USA). Helium was used as a carrier gas at a constant flow of 1 mL/min. The injection was done in splitless mode (purge time: 60 s) at 280 °C. The oven temperature program was as follows: held at 100 °C for 1 min, ramped to 280 °C at 30 °C/min, and held for 8.0 min. The total running time was 15 min. The MS transfer line was maintained at 280 °C. Mass spectrometry parameters included electron ionization at 70 eV, ion source temperature of 230 °C, and MS quadrupole temperature of 150 °C. The detector worked in multiple reaction mode (MRM) and both transitions and retention time of the compounds could be checked in Table 1. Method optimization and validation parameters can be found elsewhere [19].

#### 2.3.2. Sample Treatment

Sample treatment was performed previously in our lab [19]. Shortly, serum samples were thawed at room temperature and mixed using vortex agitation. Then, 1.5 mL of each sample was transferred to a clean vial, and 30 μL of β-glucuronidase solution (20,000 U/mL in 1 M ammonium acetate buffer, pH 5.0) was added. Samples were incubated overnight at 37 °C to allow hydrolysis. After cooling to 4 °C, 1.5 mL of acetonitrile was added. Each sample was then fortified with 20 μL of an internal standard mixture (BPAd16, BPS 13C12 and BPF 13C6) and allowed to stand at room temperature for 10 min after vortex mixing and centrifugation at 3500 rpm for 5 min. Subsequently, 1 mL of the supernatant was transferred to a clean vial, and 85 μL of tetrachloroethylene and 100 μL of acetic anhydride were added. In a conical-bottomed glass tube, 3 mL of deionized water and 300 μL of potassium carbonate (K_2_CO_3_) were added to achieve a pH ≥ 10. The mixture was quickly transferred to the glass tube and vortexed. The samples were then centrifuged at 2100 rpm for 4 min. Finally, 70 μL of the lower (organic) phase was transferred to a 100 μL insert, and 1 μL was injected into the GC-QqQ system.

#### 2.3.3. Reagents and Chemicals

BPA (≥99.0% purity), BPAF (≥99.0% purity), BPF (≥98.0% purity), BPS (≥98.0% purity), BPB (≥98.0% purity), acetonitrile (gradient grade for HPLC), acetic anhydride (>99%), tetrachloroethylene (>99%), K_2_CO_3_ and β-glucoronidase solution (20,000 U/mL in 1 M ammonium acetate buffer pH 5.0) were purchased from Sigma-Aldrich (West Chester, PA, USA). BPAd16 was purchased from LGC Standards (Teddington, UK) while BPS 13C12 was obtained from Cambridge Isotope Laboratories (Tewksbury, MA, USA) and BPF 13C6 was obtained from TRC Canada (Toronto, ON, Canada).

### 2.4. Determination of Genomic and Chromosomal Damage

To determine the levels of DNA damage present in CKD patients, two complementary assays were used, the comet assay to detect mainly DNA strand breaks and the micronucleus assay to detect chromosome breaks and aneuploidy.

- Comet (Single-Cell Gel Electrophoresis—SCGE) assay

DNA damage in peripheral blood lymphocytes was assessed using the alkaline comet assay, following a standard protocol [32]. The comet assay was performed using Gelbond^®^ films (GF) (Lonza, Basel, Switzerland) instead of traditional microscope slides, as the support for the agarose gel [33,34], increasing the efficiency of the technique.

Lymphocytes were isolated using a Ficoll-Paque density gradient from 500 μL of whole blood for each patient and were cryopreserved in 500 μL of a medium containing 90% serum and 10% DMSO until use.

Cells were adjusted to a concentration of 17,800 cells in 25 μL of PBS and carefully resuspended in 225 μL of 0.75% low-melting-point agarose (LMA) at 37 °C, then dropped onto a GF film (10.5 cm × 37.5 cm). Forty-eight drops (7 μL each) were placed on each GF, and samples from eight individuals were run simultaneously, with six drops per individual. The lymphocytes were lysed for at least 1 h at 4 °C in a dark chamber containing freshly prepared, cold lysis solution (2.5 M NaCl, 100 mM Na_2_EDTA, 10 mM Tris-HCl and 1% Triton X-100, adjusted to pH 10). To allow DNA unwinding and exposure to alkali-labile sites, the GFs were placed in a horizontal electrophoresis tank filled with fresh cold (4 °C) electrophoresis buffer (1 mM Na_2_EDTA and 300 mM NaOH adjusted to pH 13) for 35 min. Electrophoresis was then carried out in the same buffer for 20 min at 1 V/cm and 300 mA. After electrophoresis, the GFs were neutralized with two 5-min washes in 1× PBS, followed by a 1-min rinse in water, then incubated overnight in 100% ethanol for fixation. The GFs were then dried and stored in the dark at room temperature until analysis. Just before microscopic analysis, GFs were stained with 20 μL of SybrGold, a fluorescent dye specific to stain DNA and RNA. Images were examined at 20× magnification using a Komet 5.5 image analysis system (Kinetic Imaging Ltd., Liverpool, UK) equipped with an Olympus BX50 fluorescence microscope. A total of 100 randomly selected cells per patient were analyzed, and the percentage of DNA in the tail was used as a measure of DNA damage. To determine the levels of oxidized DNA bases in lymphocytes, the GFs were washed twice (10 and 50 min, 4 °C) after cell lysis in an enzyme buffer solution (40 mM HEPES, 0.1 M KCl, 0.5 mM EDTA, 0.2 mg/mL BSA, pH 8.0) containing FPG (formamidopyrimidine-DNA glycosylase) enzyme. Each sample was analyzed using two GFs. One GF remained in the cell lysis solution to assess baseline DNA damage, the second was treated with enzyme buffer without FPG to control for any buffer-only effects. GFs were incubated with enzyme buffer (with and without FPG) for 30 min at 37 °C. After incubation, the samples were processed according to the standard alkaline comet assay protocol. Net oxidative DNA damage was calculated by subtracting the damage measured in samples treated with enzyme buffer alone from the damage measured in samples treated with FPG.

- Micronucleus assay

This assay measures the frequency of micronuclei (MN, which are small chromatin bodies resulting from the condensation of chromosome fragments or whole chromosomes lagging during cell division). The relevance of MN estimations relies on the fact that are considered very sensitive indicators of genetic damage and as a good surrogate biomarker of cancer risk [30]. For the assay, whole blood cultures were prepared by adding 0.5 mL of whole blood to 4.5 mL of RPMI 1640 medium supplemented with 15% heat-inactivated fetal bovine serum, 1% antibiotics (penicillin and streptomycin), and 1% L-glutamine (all from Gibco Life Technologies, Paisley, UK). Lymphocytes were stimulated with 1% phytohemagglutinin (Gibco) and incubated at 37 °C. After 44 h, 6 μg/mL of cytochalasin B (Cyt-B; Sigma, St. Louis, MO, USA) was added to the cultures to inhibit cytokinesis. At 72 h, cultures were harvested and centrifuged at 120× *g* for 8 min. Blood cultures then underwent a gentle hypotonic treatment (2–3 min in 0.075 M KCl at 4 °C). Cells were centrifuged and fixed in a methanol/acetic acid solution (3:1 *v*/*v*). Two or more coded microscope slides were prepared and stained with 10% Giemsa (Merck, Darmstadt, Germany) in phosphate buffer (pH 6.8) for 10 min.

BNMN frequency and total MN number were assessed by blinded scoring of 1000 binucleated cells per subject (500 per replicate) with intact cytoplasm using coded slides. Additionally, 500 lymphocytes were examined to determine the percentage of cells with one, two, or more nuclei, and the cytokinesis-block proliferation index (CBPI) was calculated.

### 2.5. Statistical Analysis

Sociodemographic, clinical, and genomic damage variables of participants were analyzed. Qualitative variables were described using absolute frequencies and percentages. Quantitative variables were described using the mean and standard deviation (SD). The Kolmogorov–Smirnov test was used to assess the normality of distributions. Changes in biomarkers and genomic damage were evaluated using the Wilcoxon test for quantitative variables. For qualitative variables, the McNemar test was used. Based on the genomic damage variables showing significant changes after the intervention, additional analyses were conducted. Stepwise, backward multivariate linear regression models were constructed to identify variables independently associated with baseline levels of comet assay results and BPAF. Variables included in the models were selected based on their clinical and bibliographic relevance. Additionally, two backward stepwise multivariate models were developed to identify variables associated with the pre-post changes in comet assay results and BPAF across the entire cohort. The same approach used in the previous models was applied. All models reported beta coefficients, 95% confidence intervals (CIs), and *p*-values. Analyses were performed using RStudio software (version 4.3.3). For all tests, statistical significance was set at *p* ≤ 0.05.

## 3. Results and Discussion

### 3.1. Characteristics of the Study Population

A total of 35 patients were initially included in the study, of whom 25 completed the follow-up period. There were 7 withdrawals due to death, 2 patients underwent kidney transplantation, and one was relocated. Among the dead patients, four died from SARS-CoV-2-related complications, and the remaining deaths were due to other causes (complicated acute abdomen, hemorrhagic shock, severe obstructive pneumopathy). Thus, the final study population had a mean age of 75 years (±14), with 56% men and 44% women, and an average body mass index (BMI) of 24 kg/m^2^ (±3.9). In 48% of patients, the underlying cause of chronic kidney disease was unknown; among those with a diagnosis, 38% had a glomerular origin.

All patients were receiving conventional hemodialysis, with a mean dialysis vintage of 40 months (range: 3–240 months), averaging 10.7 (±1.6) h of dialysis per week. The predominant vascular access was a central venous catheter in 68% of cases, and 96% of patients used a VitaPES dialyzer (PALEX Medical, Barcelona, Spain), matched to their body surface area. Residual urine output, defined as >500 mL/day, was preserved in 24% of individuals. Among patients, 96% were hypertensive, 32% had diabetes, 76% had dyslipidemia, and 24% reported current tobacco use. Twelve patients (48%) had a history of cardiovascular disease, with ischemic heart disease being the most common form (60%), followed by peripheral vascular disease (20%) and stroke. A history of malignancy was present in 40% of patients, with urothelial cancer being the most frequent (30%), followed by prostate cancer (20%) and breast cancer (20%). Lastly, 24% of the population had a prior history of kidney transplantation. This information is summarized in Table 2.

Biochemical analysis of baseline and post-follow-up samples (first vs. second samples) revealed no significant differences in laboratory parameters related to anemia management, mineral bone metabolism, or nutritional status (see Supplementary Materials).

As previously indicated, CKD prevalence has increased notably over the past few decades, likely due to improved survival rates of chronic conditions such as diabetes mellitus and hypertension, as well as population aging. The leading global cause of CKD is diabetic kidney disease, followed by atherosclerotic vascular disease associated with hypertension and glomerular disorders [18]. Predictably, these factors are relevant in the study population. Charlson comorbidity index, which is an easy and valid method for estimating risk of death from comorbid disease, was also elevated in our population of patients [35,36].

### 3.2. Bisphenol Levels in the Blood of HD-CKD Patients Included in the Study

**Figure 1:**
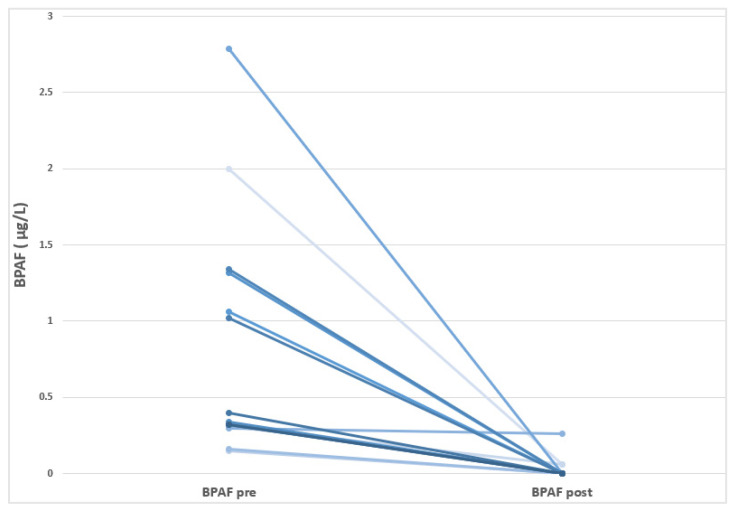
Visualization of the individual decreases in the BPAF levels in HD-CKD patients before and after moving to use BPA-free dialyzer.

In this study, we reported for the first time a wide presence of different BPs in HD-CKD patients, although its incidence differs according to each BP analogue. Thus, BPA levels were detected in 98% of the individuals sampled, while BPF could be quantified in 97% of the patients; In addition, BPAF was observed in a small number of patients (47%). The other studied BPs (BPB and BPS) showed levels under the limit of detection. As observed in Table 3, BPF showed the highest baseline concentration, with a mean of 0.97 ± 1.41 µg/L, followed by BPA (0.50 ± 0.21 µg/L), and BPAF (0.46 ± 0.75 µg/L). In the pre- and post-follow-up comparative analysis, a significant reduction in BPAF levels was observed (*p* = 0.025), with no significant changes noted for the remaining BP analogues or the total BP concentration. At the individual level, all patients with detectable baseline BPAF levels exhibited a marked decrease by the end of the follow-up period. Figure 1 shows a reduction in BPAF levels following the transition of HD-CKD patients to BPA-free dialyzers. It should be noted that BPAF is a fluorinated organic compound that is an analogue of BPA in which the two methyl groups are replaced with trifluoromethyl groups. It is important to highlight that the BPAF content (or that of any other bisphenol compound other than BPA) in the dialyzers remains unknown, as the manufacturer is not required to disclose this information.

In contrast to our study, which analyzed a broad spectrum of BPs, most investigations have only focused exclusively on BPA. Thus, it was initially demonstrated that the chronic (3-month) use of polysulfone dialyzers with BPA did not significantly increase predialysis serum BPA levels; however, the use of BPA-free dialyzers reduced predialysis serum BPA levels [37]. A subsequent study by the same group further demonstrated that switching from polysulfone to BPA-free dialyzers contributed to lowering BPA levels in dialysis patients [7]. In our study, however, no significant reduction in BPA levels was observed following the introduction of BPA-free dialyzers. This discrepancy may be due to other concurrent sources of exposure, the pharmacokinetics of BPA, or the limited sample size and variability across patients. Overall, these findings underline the complexity of BPA exposure in hemodialysis patients and underscore the need of larger studies that account for multiple exposure pathway.

### 3.3. Genomic Damage in HD-CKD Patients Before and After Moving to Use BP-Free Dialyzers

In addition to the internal exposure to BPs, DNA damage is another potential health effect associated with xenobiotic exposures. The relevance of such effects is because genotoxicity is a well-established mechanism linked to cancer development [38]. According to the relevance of DNA damage as a biomarker of effect, in this study we used two complementary genotoxicity assays: comet and micronucleus assay. The first, mainly determines DNA single strand breaks and alkali-labile sites as indicators of primary DNA damage [24]. This means that this type of primary DNA damage can be recognized by the repair machinery and be properly repaired. The micronucleus assay detects chromosome breaks and aneuploidy and, consequently, the observed fixed effects are strong indicators of effects altering DNA structure and organization [39]. Since most studies assessing the genotoxicity of BPs have focused on BPA, a substantial body of evidence on its DNA damaging potential has been generated and was recently reviewed [40]. To sum up, the authors pointed out the large variability of reported data suggesting that the genotoxicity of BPA depends on different factors such as cell type, the duration of exposure, and BPA concentration.

**Figure 2:**
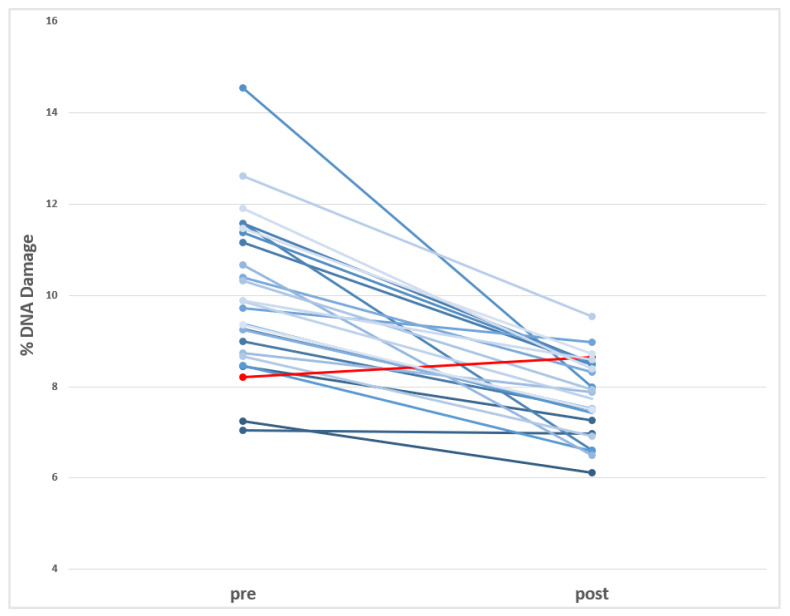
Visualization of the individual decreases in the levels of DNA damage (Comet assay) observed in HD-CKD patients before and after using BPA-free dialyzer. (in red is indicated the only donor showing increases after using BPA-free dialyzer).

The effects observed in this study using the comet assay are presented in Table 4. As shown, significant variations are detected in direct DNA strand breaks before and after the switch to BPA-free dialyzers. Nevertheless, the levels of oxidized DNA bases are not modified by the switch in the HD process. These changes are visualized in Figure 2, where the individual data (before and after the dialyzer switch) are represented.

Although DNA damage in HD-CKD patients has been the topic of multiple studies [41], its association with BPs has never been determined, highlighting the novelty of our study. Nevertheless, the role of HD characteristics on the DNA damage levels has already been determined looking for the relevance of using vitamin E-coated membranes on the DNA damage levels. Significant decrease in the levels of oxidative DNA damage was observed after using vitamin E-coated membranes, in addition of improving the uremic anemia status of the patients [42]. Similar effects were also observed in patients who switched from standard HD to online-hemodialysis filtration showing a significant reduction in the levels of DNA damage [43].

The effects detected by the comet assay were not confirmed by the micronucleus assay. This discrepancy is not unexpected, as both assays measure different genetic endpoints. While the comet assay is sensitive to transient DNA strand breaks, the micronucleus assay detects fixed chromosomal, making it a more restrictive biomarker of incidence, but one with greater relevance to long-term health effects. As indicated in Table 5, the apparent increases in the post-sampling are not significantly different, indicating that the levels of chromosome alterations (both structural and numeric) are not affected by moving HD patients from normal to BP-free dialyzers. Despite these negative findings, micronuclei are considered a good biomarker of CKD status since patients in the predialysis or hemodialysis state of the disease exhibit higher levels of genomic damage, measured as micronucleus frequency in peripheral blood lymphocytes and buccal mucosa cells, than healthy control groups [39].

### 3.4. Factors Modulating the DNA Damage Decrease (Comet Assay) When Moving to BP-Free Dialyzers

In the stepwise regression model for the reduction in baseline genomic damage levels (Table 6), it was observed that patients with higher initial levels showed a more significant decrease by the end of the follow-up period (*p* ≤ 0.001). Similarly, individuals who had been on dialysis for more than 24 months or had C-reactive protein (CRP) levels above 5 mg/L (with more inflammation) also showed a greater reduction in baseline genomic damage (*p* = 0.017 and *p* = 0.054, respectively). In contrast, patients with a history of cardiovascular disease exhibited a significantly smaller reduction in baseline damage (*p* = 0.05), as did individuals with high comorbidity scores (Charlson index), although this latter finding did not reach a level of statistical significance (*p* = 0.07).

In HD-CKD patients, multiple factors may contribute to the observed increase in genomic damage. These include advanced age, underlying comorbidities, dialysis technique, recurrent infections, and intravenous treatments such as iron therapy. The clinical consequences of elevated genomic damage are well established. Thus, Coll et al. evaluated mortality in 123 hemodialysis patients and found that those with higher levels of genomic damage had significantly higher mortality. Only age, inflammation, and oxidative genomic damage (measured by EndoIII, that is an enzyme treatment that permits to determine the net effect on pyrimidines) were identified as independent predictors of mortality [44]. Accordingly, various therapeutic strategies have been explored to reduce genomic damage. For instance, supplementation with fermented grape juice (must), which is rich in flavonoids, was studied in 25 hemodialysis patients compared to a control group. A significant reduction in oxidative damage was observed in the supplemented group [45]. Another approach involved the change in polysulphone dialysis membranes by other coated with vitamin E (a known antioxidant), which showed a significant decrease in oxidative damage compared to the control group, particularly notable in patients who had very high baseline genomic damage [42].

It must be pointed out that in the performed multivariate analysis, the observed variation in BPAF levels did not correlate with any clinical, therapeutic, or analytical variables, suggesting that the changes may be attributable to the intervention performed during the study. Since the manufacturer does not specify the content of different BP analogues in the dialyzers, a combined analysis of all of them was conducted, treating them as markers of microplastic presence. This analysis also did not reveal any significant differences. It is likely that measuring BPs represents only the tip of the iceberg in a much broader issue, the interaction between living organisms and environmental micro- and nanoplastics (MNPLs), primarily through their entry into the food chain. Various compounds or additives found in MNPLs have been identified as having potential toxic effects. However, it is also likely that many more molecules remain unidentified or that their biological impact is still unknown, much like what is observed with uremic toxins. Among such plastic additives, BPs stand out in the literature. BPs are reported to induce a wide set of harmful effects, including DNA damage [46] while their widespread distribution is translated to a global human exposure that can differ according to country and population characteristics [47]. At the general population level some studies have explored the potential associations between exposure levels and genotoxicity biomarkers. According to their role as endocrine disruptors, because they can act as xenoestrogens [48], most of the studies focus on factors associated with reproductive outcomes. Thus, the levels of fifteen BPs, were associated with the DNA oxidative damage levels, as measured using as a biomarker the presence of 8-hydroxy-2-deoxyguanosine (8-OHdG) in the urine of pregnant women recruited in south China. The levels of 8-OHdG were positively correlated with the urinary concentrations of several bisphenol analogues [49]. In addition, repeated measurements of urinary BPA, BPF, and BPS concentrations in Chinese men were associated with increased levels of DNA damage in sperm, as determined with the comet assay. Interestingly, such association was more pronounced in men with low body mass index and non-smokers [50].

Collectively, these findings support an association between elevated levels of BPs, increased levels of DNA damage, and adverse health outcomes. Although the widespread environmental presence of BPs affects all human populations, HD-CKD patients must be considered as a very sensitive group. The inability to efficiently eliminate BPs, combined with the risk of extracorporeal contamination, likely amplifies their internal burden and associated genotoxic effects.

## 4. Conclusions

In our cohort of hemodialyzed patients, switching to BPA-free dialyzers resulted in a significant reduction in BPAF levels. This change was also accompanied by a marked decrease in DNA damage, as measured by the comet assay. These results underscore the importance of removing not only BPA but also other BP analogues from dialyzers and related medical devices used in hemodialysis. To date, no studies have evaluated the in vivo genotoxic potential of bisphenols in humans, highlighting the novelty and relevance of our results. Therefore, due to the characteristics of the disease, the hemodialysis population constitutes a highly representative model for studying this relationship, and our study is the first to evaluate the possibility of reducing genomic damage by reducing exposure to bisphenols in patients with CKD on hemodialysis. Our findings contribute to strengthening evidence for the need to replace materials such as bisphenols in the manufacture of medical devices, especially those used in hemodialysis, trying to reduce the high morbidity and mortality rates experienced by this population.

## Figures and Tables

**Table 1:** Retention time and transitions of the compounds analyzed.

Compound	Retention Time (min)	Quantitative Transition (*m*/*z*)	Qualitative Transition (*m*/*z*)	Qualitative Transition (*m*/*z*)
BPAF	7.218	378 → 336	267 → 227	336 → 267
BPF 13C6	7.853	206 → 113	248 → 206	206 → 107
BPF	7.857	200 → 107	242 → 107	200 → 94
BPAd16	8.215	242 → 224	242 → 125	224 → 97
BPA	8.256	228 → 213	228 → 119	213 → 91
BPB	8.653	213 → 119	213 → 91	255 → 119
BPS 13C12	10.151	304 → 262	262 → 147	262 → 116
BPS	10.155	250 → 141	250 → 110	292 → 110

**Table 2:** Descriptive of the study population.

*n* = 25	Mean (SE)	Range
Age (years)	75 (14)	39–92
Weight (kg)	65.61 (11.07)	42–90
Height (m)	1.65 (0.08)	1.49–1.79
Body mass index (kg/m^2^)	24.16 (3.90)	15–30
HD time (months)	40.96 (47.88)	3–240
Hours per week HD	10.71 (1.65)	6–12
Charlson comorbidity index	7.32 (2.36)	3–10
***n = 25***	**Yes**	**No**
Previous kidney transplant	6 (24%)	19 (76%)
Diuresis residual	6 (24%)	19 (76%)
Smoking active	6 (24%)	19 (76%)
Ex-smoking	11(44%)	14 (56%)
High blood pressure	24 (96%)	1 (4%)
Diabetes mellitus	8 (32%)	17 (68%)
Dyslipidemia	19 (76%)	6 (24%)
Cardiovascular Disease	12 (48%)	13 (52%)
Previous neoplasms	10 (40%)	15 (60%)
Catheter/Fistula	17 (68%)	8 (32%)

**Table 3:** BP concentration in blood of HD-CKD patients before and after moving to use BPA-free dialyzer.

n: 25		First Sample	Second Sample	*p*-Value
BPA (µg/L)	Mean (SD)	0.50 (0.21)	0.71 (0.68)	0.554
	Range	0.14–0.95	0.16–3.20	
BPAF (µg/L)	Mean (SD)	0.46 (0.75)	0.14 (0.61)	0.025 *
	Range	0.01–2.79	0.01–3.13	
BPF (µg/L)	Mean (SD)	0.97 (1.41)	0.92 (0.96)	0.439
	Range	0.01–6.50	0.00–4.24	
BPB (µg/L)	Mean (SD)	0.01 (0.00)	0.05 (0.23)	1
	Range	0.01–0.01	0.01–1.17	
BPS (µg/L)	Mean (SD)	0.10 (0.48)	0.18 (0.41)	0.855
	Range	0.01–2.44	0.01–1.22	
ALL BPs (µg/L)	Mean (SD)	1.93 (1.39)	1.77 (1.61)	0.6
	Range	0.29–6.80	0.45–7.45	

* Wilcoxon test.

**Table 4:** DNA damage levels in HD-CKD patients before and after moving to use BPA-free dialyzers. Damage without (basal) and with FPG enzyme (oxidative) are indicated as obtained using the comet assay.

n: 25		First Sample	Second Sample	*p*-Value *
DNA damage	Mean (SD)	10.01 (1.71)	7.79 (0.86)	<0.001
	Range	7.04–14.54	6.12–9.54	
Oxidative DNA damage	Mean (SD)	4.99 (1.99)	5.72 (2.60)	0.237
	Range	1.67–8.53	1.79–14.79	

* Wilcoxon test.

**Table 5:** Chromosomal damage levels in HD-CKD patients before and after moving to use BPA-free dialyzers. Damage is determined by the micronucleus test.

n: 25		First Sample	Second Sample	*p*-Value *
MN	Mean (SD)	10.20 (5.66)	12.69 (10.35)	0.361
	Range	4.00–25.00	2.00–46.00	
BNMN	Mean (SD)	9.44 (5.25)	11.35 (3.40)	0.376
	Range	4.00–25.00	2.00–34.00	
CBPI	Mean (SD)	1.50 (0.18)	1.55 (0.16)	0.157
	Range	1.18–1.89	1.19–1.81	

MN: micronuclei: BNMN: micronuclei in binucleated cells, CBPI: cytokinesis-block proliferation index. * Wilcoxon test.

**Table 6:** Stepwise linear regression model detecting factors associated with the observed decrease in the levels of DNA damage (comet assay).

n: 25	Coefficient	LI IC 95%	LS IC 95%	*p*-Value
Initial DNA damage levels	0.85	0.61	1.09	**<0.001**
Time in HD (≥24 months)	1.04	0.23	1.84	**0.017**
Hour/week in HD (≥12 h)	−0.35	−1.01	0.32	0.268
Diabetes (Si)	1.13	0.30	1.96	**0.013**
Neoplasia (SI)	−0.50	−1.21	0.21	0.145
ECV (Si)	−0.74	−1.52	−0.04	**0.050**
Kt/V (≥1.3)	0.34	−0.37	1.05	0.309
CRP (≥5)	0.69	−0.01	1.39	**0.054**
Pre-albumin	−3.40	−9.31	2.52	0.226
Charlson index ≥ 8	−0.71	−1.51	0.08	0.073

HD: hemodialysis, ECV: cardiovascular disease, CRP: C reactive protein.

## Data Availability

The original contributions presented in this study are included in the article/Supplementary Materials. Further inquiries can be directed to the corresponding author(s).

## Supplementary Materials

The following supporting information can be downloaded at https://www.mdpi.com/article/10.3390/jox15050167/s1, Table S1. Analysis of Anaemia follow up in HD-CKD patients before and after moving to use BPA-free dialyzer; Table S2. Analysis of mineral and bone metabolism in HD-CKD patients before and after moving to use BPA-free dialyzer; Table S3. Analysis of nutritional parameters in HD-CKD patients before and after moving to use BPA-free dialyzer.
