# Supplementary material for: DNA Damage and Bisphenol Levels in Chronic Kidney Disease Patients Undergoing Hemodialysis
Source: J Xenobiot. 2025 Oct 17;15(5):167. doi: 10.3390/jox15050167 (PMC12565573; doi:10.3390/jox15050167)
Supplement: Supplementary file 1 [file jox-15-00167-s001.zip › jox-3798898-supplementary.pdf]

Journal of Xenobiotics

MDPI

Supplementary Materials: DNA Damage and Bisphenol Levels in Chronic Kidney Disease Patients Undergoing Hemodialysis

Cesar Emilio Ruiz, Lourdes Vela, Martí Nadal, Neus González, Ricard Marcos, Alba Hernández, Susana Pastor and Elisabeth Coll

Table S1. Analysis of Anaemia follow up in HD-CKD patients before and after moving to use BPA-free dialyzer

**n: 25**		**First sample**	**Second sample**	***p-value***
IV Fe	No	10 (40.0%)	4 (20.0%)	*0.45*
	Yes	15 (60.0%)	16 (80.0%)	
Type of IV Fe	Sucrose	15 (100.0%)	14 (93.3%)	*n/a*
	Carboxymaltose	0 (0.0%)	1 (6.7%)	
Weekly iron supplementation (mg)	Mean (SD)	69.40 (35.91)	110.84 (232.70)	*0.272*
EPO	Yes	0 (0.0%)	1 (4.8%)	*n/a*
	No	25 (100.0%)	20 (95.2%)	
EPO/week (mcg)	Mean (SD)	56.60 (37.21)	58.00 (48.41)	*0.864*
Hb (g/L)	Mean (SD)	114.68 (20.09)	115.48 (14.95)	*0.881*

IV Fe: intravenous iron, EPO: erythropoietin, Hb: haemoglobin, n/a: not applicable.

Test: Wilcoxon. SD: standard deviation

Table S2. Analysis of mineral and bone metabolism in HD-CKD patients before and after moving to use BPA-free dialyzer

	n: 25	First sample	Second sample	*p-value*
Calcium (mmol/L)	Mean (SD)	2.23 (0.16)	2.22 (0.19)	*0.928*
	Range	1.74 - 2.50	1.97 - 2.67	
Phosphorus (mmol/L)	Mean (SD)	1.42 (0.43)	1.39 (0.47)	*0.689*
	Range	0.69 - 2.35	0.78 - 2.61	
PTH (ng/L)	Mean (SD)	196.02 (145.77)	238.47 (193.34)	*0.236*
	Range	18.00 - 702.00	2.50 - 787.00	
Calcidiol (ng/L)	Mean (SD)	26.95 (25.00)	31.00 (23.24)	*0.359*
	Range	1.60 - 94.00	9.60 - 84.00	

PTH: parathyroid hormone.

Test: Wilcoxon. SD: standard deviation

Table S3. Analysis of nutritional parameters in HD-CKD patients before and after moving to use BPA-free dialyzer.

**n: 25**		**First sample**	**Second sample**	***p-value***
Albumin (g/L)	Mean (SD)	39.45 (4.32)	38.90 (4.66)	*0.367*
	Range	29.2 - 48.5	22.2 - 45.6	
Proteins (g/L)	Mean (SD)	67.90 (7.43)	66.58 (5.91)	*0.253*
	Range	49.00, 81.80	52.8 - 78.9	
Prealbumin (g/L)	Mean (SD)	0.25 (0.06)	0.28 (0.06)	***0.016*** *
	Range	0.12 – 0.37	0.17 – 0.39	
Cholesterol (mmol/L)	Mean (SD)	3.71 (0.93)	3.65 (0.78)	*0.684*
	Range	2.47 - 6.51	2.49 - 5.24	
LDL (mmol/L)	Mean (SD)	1.91 (0.78)	1.92 (0.61)	*0.877*
	Range	0.78 - 4.16	0.97 - 3.36	
HDL (mmol/L)	Mean (SD)	1.13 (0.31)	1.07 (0.32)	*0.191*
	Range	0.69 - 1.82	0.54 - 1.86	
TG (mmol/L)	Mean (SD)	1.51 (0.70)	1.55 (0.92)	*0.966*
	Range	0.52 - 3.80	0.60 - 4.86	

LDL: low-density lipoprotein, HDL: high-density lipoprotein, TG: triglycerides.
Test: Wilcoxon. SD: standard deviation
